# Verification of the Usefulness of an Assessment and Risk Control Sheet that Promotes Management of Cancer Drug Therapy

**DOI:** 10.3389/fphar.2022.744916

**Published:** 2022-02-09

**Authors:** O. Honma, C. Watanabe, H. Fukuchimoto, J. Kashiwazaki, M. Tateba, S. Wagatsuma, K. Ogata, K. Maki, H. Sonou, K. Shiga, E. Otsuka, M. Hiruta, Y. Hirasawa, M. Hosonuma, M. Murayama, Y. Narikawa, H. Toyoda, T. Tsurui, A. Kuramasu, M. Kin, Y. Kubota, T. Sambe, A. Horiike, H. Ishida, K. Shimada, M. Umeda, T. Tsunoda, K. Yoshimura

**Affiliations:** ^1^ Department of Nursing, Showa University Hospital, Tokyo, Japan; ^2^ Department of Nursing, Showa University School of Nursing and Rehabilitation Sciences, Kanagawa, Japan; ^3^ Department of Nursing, Showa University Koto Toyosu Hospital, Tokyo, Japan; ^4^ Faculty of Nursing, Kyoritsu Women’s University, Tokyo, Japan; ^5^ Department of Medical Oncology, Showa University, Tokyo, Japan; ^6^ Department of Clinical Immunology and Oncology, Clinical Research Institute for Clinical Pharmacology and Therapeutics, Showa University, Tokyo, Japan; ^7^ Department of Pharmacy, Showa University Hospital, Tokyo, Japan; ^8^ Division of Clinical Pharmacology, Department of Pharmacology, Showa University School of Medicine, Shinagawa-ku, Japan; ^9^ Division of Medical Oncology, Showa University Northern Yokohama Hospital, Yokohama, Japan; ^10^ Division of Medical Oncology, Internal Medicine Center, Showa University Koto Toyosu Hospital, Tokyo, Japan; ^11^ Family Hospice Co., Ltd., Tokyo, Japan

**Keywords:** drug therapy, adverse event, common terminology criteria for adverse events, nursing, assessment, pre-examination

## Abstract

**Background:** Proper management of adverse events is crucial for the safe and effective implementation of anticancer drug treatment. Showa University Hospital uses our interview sheet (assessment and risk control [ARC] sheet) for the accurate evaluation of adverse events. On the day of anticancer drug treatment, a nurse conducts a face-to-face interview. As a feature of the ARC sheet, by separately describing the symptoms the day before treatment and the day of treatment and sharing the information on the medical record, it is possible to clearly determine the status of adverse events. In this study, we hypothesized that the usefulness and points for improvement of the ARC sheet would be clarified by using and evaluating a patient questionnaire.

**Methods:** This study included 174 patients (144 at Showa University Hospital (Hatanodai Hospital) and 30 at Showa University Koto Toyosu Hospital (Toyosu Hospital) who underwent pre-examination interviews by nurses and received cancer chemotherapy at the outpatient center of Hatanodai and Toyosu Hospital. In the questionnaire survey, the ARC sheet’s content and quality, respondents’ satisfaction, structural strengths, and points for improvement were evaluated on a five-point scale.

**Results:** The patient questionnaire received responses from 160 participants, including the ARC sheet use group (132 people) and the non-use group (28 people). Unlike the ARC sheet non-use group, the ARC sheet use group recognized that the sheet was useful to understand the adverse events of aphthous ulcers (*p* = 0.017) and dysgeusia (*p* = 0.006). In the satisfaction survey questionnaire, there was a high sense of security in the pre-examination interviews by nurses using the ARC sheet.

**Conclusions:** The ARC sheet is considered an effective tool for comprehensively evaluating adverse events. Pre-examination interviews by nurses using ARC sheets accurately determined the adverse events experienced by patients with anxiety and tension due to confrontation with physicians.

## Introduction

Cancer drug therapy is effective in controlling tumor progression, relieving symptoms, and prolonging survival. However, it is often associated with adverse events. The proper use of drugs and management of adverse events are crucial for the safe and effective implementation of cancer drug therapy. Adverse events in patients undergoing cancer drug therapy may include subjective symptoms, many of which are not identified without asking the patient directly. In clinical practice, these symptoms are checked during medical examination and nursing care. The National Cancer Institute’s Common Terminology Criteria for Adverse Events (NCI-CTCAE) is a set of criteria used for the standardized classification of adverse events. However, even if patients want to report adverse events or symptoms of concern during a doctor’s visit, they cannot communicate everything in the limited time available during the visit, and patients tend to focus on the symptoms they are concerned about, resulting in a discrepancy between the patient’s subjective assessment and the doctor’s assessment of symptoms (Basch, 2010; Baratelli et al., 2019; Liu et al., 2020). Since the 1990s have seen increasing attention to patient-reported outcomes, and the National Cancer Institute has developed the Patient-Reported Outcomes version of the Common Terminology Criteria for Adverse Events (PRO-CTCAE) to enable patients to self-report their adverse events (Basch et al., 2017). The PRO-CTCAE has been used to evaluate symptomatic toxicity in patients on cancer clinical trials (Basch et al., 2016; Basch, 2017; Basch et al., 2017; Kawaguchi et al., 2017; Baratelli et al., 2019; Wong et al., 2019). One study has reported that a 5-month extension in the overall survival time by using the PRO-CTCAE (Basch et al., 2017). It works by evaluating the most severe symptoms of one adverse event in the past week based on the patient’s answers to multiple questions regarding frequency, severity, and effects on daily life (https://healthcaredelivery.cancer.gov/pro-ctcae/item-library.pdf). Although it is possible to determine the degree and severity of symptoms in the last week, it is insufficient to identify the onset of adverse events according to different schedules for each regimen and the degree of physical and psychological distress that occurred at that time.

In an attempt to ascertain adverse events prior to outpatient anticancer treatment, the physician may interview the patient during the consultation, or the patient may fill out a questionnaire on their own, or the nurse may conduct an interview. Another method is for the nurse to conduct a telephone interview at intervals of several days after treatment (Cirillo et al., 2009; Traeger et al., 2015; Bayraktar-Ekincioglu and Kucuk, 2018; Kotronoulas et al., 2018).

Therefore, on the day of anticancer drug treatment, before the patient was examined by the doctor, a nurse attempted to ascertain adverse events in detail by conducting a face-to-face interview with the patient in a separate room using an ARC (Assessment and Risk Control) sheet. The ARC sheet is designed to assess changes in physical condition at intervals of one to 4 weeks between the date of the previous treatment and the date of the current treatment, and the interviews are conducted on the day before and the day of the treatment.

After the nurse conducts the interview, information in electronic medical records can be shared with physicians and pharmacists so that the medical team can determine the adverse events precisely. A further noteworthy advantage of the ARC sheet is that nurses can conduct face-to-face pre-examination interviews with patients worried that their adverse events will not be accurately communicated during physical examination. Further, by using the ARC sheet, patients can report the adverse events they experienced while waiting for the examination. Because ARC sheets are immediately shared on the electronic medical record, they help physicians, nurses, and pharmacists to collaborate in managing a diverse range of adverse events.

We aimed to use the ARC sheet to more accurately and broadly identify adverse events that patients are unable to communicate through physician interviews alone, and to share this information with the medical team. In order to investigate whether this ARC sheet actually has the intended function, we examined the following points. First, we aimed to identify particular strengths and areas for improvement of the ARC sheet by surveying patients using five segments. The five segments are: “Ease of communicating adverse events in treatment,” “Ease of communication with medical staff,” “Subject of interview,” “Reassurance about nurse’s response,” and “System of pre-inspection interview”. These five segments are intended to provide an accurate picture of the content and quality of the ARC sheet, the level of satisfaction of the respondents, and the structural strengths and areas for improvement. Ultimately, the ARC sheet is intended to be used worldwide as a universal and easy-to-use platform for healthcare teams to share information on adverse events when administering cancer medications.

## Methods

### Patients

This study comprised 144 patients who received outpatient cancer drug therapy in the Department of Oncology, Hatanodai Hospital, from October 2019 to July 2020, and Showa University Koto Toyosu Hospital from January 2020 to March 2020. Moreover, 30 patients who received outpatient cancer drug therapy in the oncology department were able to undergo a questionnaire survey. A total of 160 patients completed the questionnaire: 132 from the Department of Oncology, Hatanodai Hospital, and 28 from the Department of Oncology, Toyosu Hospital . The patients’ responses were analyzed.

### Details of the Survey

The ARC sheet contains seven items of objective data on body temperature, pulse rate, blood pressure, respiratory rate, oxygen saturation, performance status, and weight, and 13 items of subjective data such as nausea, vomiting, loss of appetite, diarrhea, constipation, skin disorders, nail disorders, peripheral nerve disorders, disability, malaise, dyspnea, and pain. Subjective data evaluate changes in physical condition at intervals of 1–4 weeks from the previous treatment day to the day of treatment. Interviews are conducted separately on the day of medical treatment and the day of treatment. The description of the subjective data was based on the evaluation of adverse events CTCAE version 5.0 (grade 0 without symptoms to grade 1–4 with symptoms). Additionally, Pain was assessed by the Numerical Rating Scale (NRS). Specifically, it is a graded scale that indicates the level of current pain, divided into 11 levels from 0 to 10, with 0 being no pain and 10 being the maximum pain imaginable. The pre-examination interview was conducted by a nurse trained in evaluating adverse events using the CTCAE. It was performed face-to-face in a room separate from the examination room. In the pre-examination interview, the patient used a self-administered notebook describing the adverse events between the treatment days. The information obtained during the interviews was promptly shared in an electronic medical record so that physicians could check on this information at the time of the examination (see Supplementary Figure S1). The physicians evaluated the adverse events by referring to the ARC sheet shared in the electronic medical record at the time of the examination.

The questionnaire survey was divided into the following five segments: 1) treatment adverse events (digestive symptoms, skin problems, tiredness/malaise, limb sensation changes, taste changes, and pain); 2) ease of communication, three items (a sense of security, ease of communicating the content of the consultation, and understanding oneself); 3) eligibility for interview, five items (accuracy of today’s physical condition change, accuracy of interval physical condition change, good information sharing by medical staff, confirmation of symptoms without omissions, and satisfaction with response time); 4) a sense of security regarding the nurse’s response, four items (awareness of potential symptoms, suggestions for coping methods, a sense of security for communicating adverse events due to pre-examination interviews, and arrangement of contents to be communicated to physicians); and 5) mechanisms for receiving pre-examination interviews, three items (effective use of waiting time, ease of pre-examination interviews, and consideration of privacy). Each item was answered on a four-point scale from “Strongly disagree” to “Strongly agree.” (see Supplementary Figures S1A–C). Regarding the medical information found in electronic medical records, the age, sex, performance status, regimen, and CTCAE grade evaluation of each participant were investigated.

### Survey Procedure

For questionnaire distribution, the physician in charge instructed the participants to complete the survey in writing. When consent was obtained, an anonymous response was requested. The completed questionnaire surveys were collected at the questionnaire survey collection box installed in both Hatanodai and Toyosu Hospital. Patients were requested to return the completed questionnaire by mail within 2 weeks.

### Statistical Analyses

JMP® Pro (version 14.0.0, 2018 SAS Institute Inc.) was used for data analysis, and the statistical significance level for all analyses was set at 5% for both. Additionally, the questionnaire survey changed the response scores from 0 to 4 to 1–5 for analysis. Descriptive statistics were performed for each variable, and a t-test was performed to compare the two groups. Receiver operating characteristic curve analysis was used to determine the optimal cutoff scores for Seg C, Seg I, Seg N, and Seg S (see Supplementary Figure S3).

### Ethical Considerations

The study’s objectives were explained to participants in writing, and their cooperation was treated with respect. We clearly emphasized that this study had no medical disadvantage regardless of whether patients chose to participate. We also pointed out that participants’ privacy and confidentiality would be strictly adhered to and that their information would be used only for the purposes of this study.

## Results

Questionnaire surveys were distributed to 144 patients at Hatanodai Hospital and 30 patients at Toyosu Hospital (Table 1), of which 132 (92%) and 28 (93%), respectively, returned a completed questionnaire (92% overall).

**TABLE 1:** Demographic data and malignancy locations.

	Hatanodai (H) Hospital	Toyosu (T) Hospital	Both hospitals (B)
Age (median, range)	66.3 (38–87)	61.7 (37–85)	64.0 (37–87)
Sex			(%)
Men	83 (57.6)	23 (76.7)	106 (60.9)
Women	61 (42.4)	7 (23.3)	68 (39.1)
Performance Status			(%)
0	8 (5.6)	—	-
1	129 (89.6)	—	-
2	7 (4.9)	—	-
Primary neoplasia			(%)
Hypopharynx	1 (0.7)	—	1 (0.6)
Esophagus	24 (16.7)	—	24 (13.4)
Lung	30 (20.8)	—	30 (16.8)
Breast	12 (8.3)	—	12 (0.06)
Stomach	21 (14.6)	5 (16.7)	26 (14.5)
Duodenum	-	1 (3.3)	1 (0.6)
Pancreas	1 (0.7)	—	1 (0.6)
Gallbladder	1 (0.7)	1 (3.3)	2 (1.1)
Colon (appendix)	18 (12.5)	13 (43.3)	31 (17.3)
Rectum	20 (13.9)	10 (33.3)	30 (16.8)
Ovary	3 (2.1)	—	3 (1.7)
Bladder	1 (0.7)	—	1 (0.6)
GIST	2 (1.4)	—	2 (1.1)
Malignant melanoma	1 (0.7)	—	1 (0.6)
Sarcoma	5 (3.5)	—	5 (2.8)
Unknown primary	2 (1.4)	—	2 (1.1)
Total	144	30	174

**TABLE 2:** The evaluation of adverse events by CTCAE up to the day before and on the day of cancer drug therapy administration (left two rows)/The patient satisfaction ratings based on patient questionnaires for the ARC sheet use group and non-use group (right two rows).

	The average grade evaluation of CTCAE		Mean level of satisfaction that each adverse event that patients wanted to communicate to medical staff was well communicated	
	The ARC sheet use group (N = 144)		The ARC sheet use group (N = 144)	The ARC sheet non-use group (N = 28)
	Until the day before the visit	On the day of visit		
Nausea	0.125	0.047	4.008	3.981
Vomiting	0.015	0.008		
Anorexia	0.203	0.156		
Diarrhea	0.18	0.117		
Constipation	0.477	0.43		
Mucositis oral	0.141	0.086		
Skin disorder	0.703	0.68	3.931	3.937
Paronychia	0.336	0.325		
Alopecia	0.469	0.477	-	-
Peripheral neuropathy	0.727	0.688	3.803	3.786
General fatigue	0.457	0.39	3.734	3.699
Dyspnea	0.25	0.219	-	-

### Characteristics and Usefulness of the ARC Sheet

The ARC sheet is a highly versatile questionnaire that can be used for a variety of regimens. The regimens used at each facility are listed in Supplementary Tables S1, S2. The ARC sheet has the feature of being able to evaluate a wide range of adverse events that are relatively frequent in cancer drug therapy. In this study, the adverse events listed on the sheet were comprehensively described regardless of the degree of CTCAE grade (Supplementary Table S3). Since the majority of adverse events are mild (grade 0–1) (Table 2), AEs may be overlooked because patients do not actively report their symptoms, but using the ARC sheet, even mild symptoms can be ascertained and detailed assessment of AEs can be performed. However, there was no apparent difference in the average grade of CTCAE, which was described separately for adverse events up to the day of examination and on that day (Supplementary Figures S4A,B). Therefore, ARC sheets are unlikely to reflect changes over time in treatment and treatment intervals in some areas.

**FIGURE 1:**
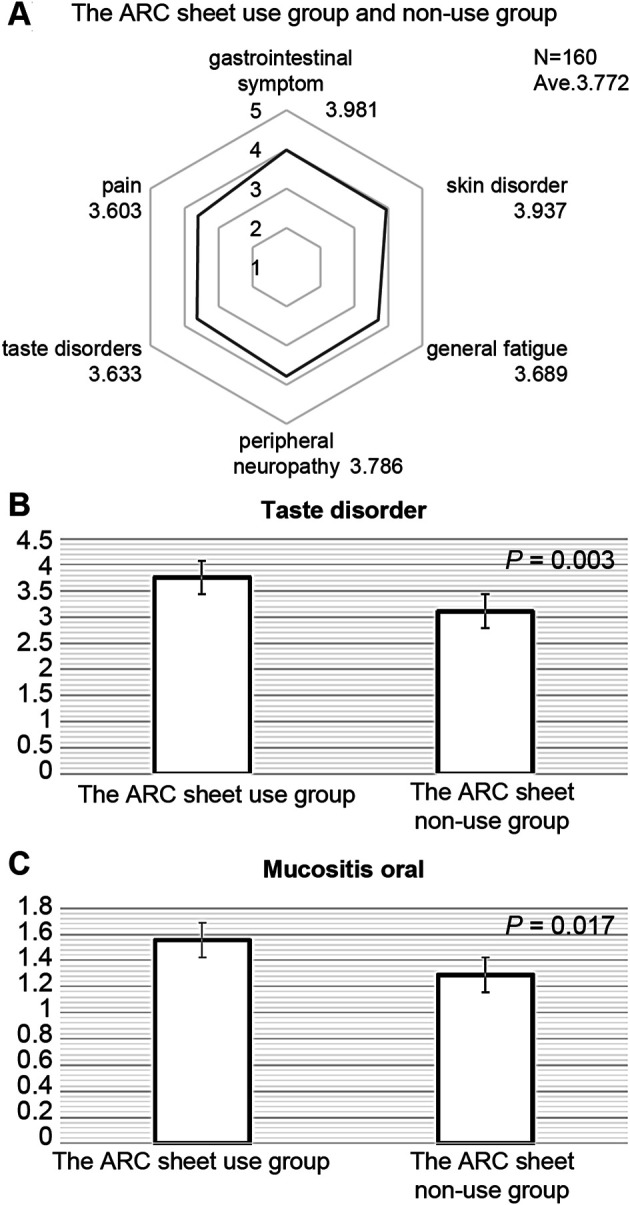
**(A)**: Adverse events in which patients in the ARC sheet use group and non-use group answered that the adverse events they wanted to communicate to medical staff were well communicated. Gastrointestinal symptoms included symptoms of nausea, decreased appetite, mouth ulcers, diarrhea, and constipation. There is no significant difference between the ARC sheet use group and the non-ARC sheet use group in terms of symptoms other than stomatitis and taste disorder. **(B)**: Comparison of satisfaction with the ease of communicating taste disorder adverse events between the ARC sheet use group and the non-use group. The use of the ARC sheet is useful in understanding taste disorder. **(C)**: Comparison of satisfaction with the ease of communicating adverse events of stomatitis between the ARC sheet use and non-use groups. The use of the ARC sheet is useful in understanding oral mucositis.

Figure 1A shows the adverse events for which patients in both the ARC sheet and non-ARC sheet groups reported that the adverse events they wanted to communicate to medical staff were well communicated. It was found that the ARC sheet helped understand the patient’s oral mucositis (*p* = 0.017) and dysgeusia (*p* = 0.003) (Figures 1B,C). For other adverse events, there was no significant difference in understanding of adverse events between the ARC sheet use and non-use groups (Table 2).

### Association Between Ease of Communication of Adverse Events and Satisfaction With Pre-Examination Interviews by Nurses or Physicians

**FIGURE 2:**
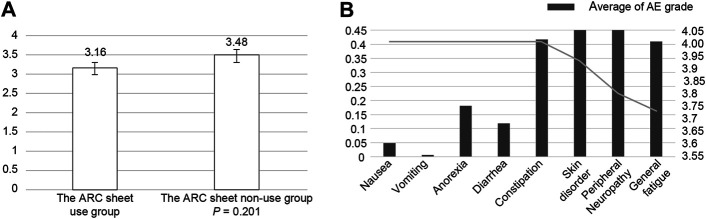
**(A)**: Patients’ satisfaction with the ability of the ARC sheet to relieve their anxiety about being able to communicate adverse events to their doctors. The ARC sheet group tended to have less anxiety about whether adverse events would be communicated, although the difference was not significant. **(B)**: In the group using the ARC sheet, the mean CTCAE grade of adverse events obtained from the ARC sheet was compared with the satisfaction with the ease of communicating adverse events obtained from the patient questionnaire. Increased adverse events affected satisfaction with pre-examination interviews.

Patients who found it easier to communicate adverse events with or without an ARC sheet were associated with ease of communication, eligibility for interviews, a sense of security for nurses, and a satisfactory pre-examination interview system (Figures 2A,B; Supplementary Figures S5A–D, S6). The mean value of satisfaction for the entire segment was 3,714. Looking at the level of satisfaction for each segment, the mean levels of satisfaction for ease of communication and eligibility for consultation were 3.903 and 3.845, respectively, which were higher than the overall mean, indicating a high level of satisfaction for these two segments (Table 3; Supplementary Figures S5A,B). On the other hand, the mean values of comfort with the nurse’s response and satisfaction with this system of pre-consultation questionnaires were 3.497 and 3.552, which were lower than the overall mean values. In relation to this, the survey suggested that there was concern that the use of ARC sheets would increase the waiting time for consultations and concern about the tediousness of being asked the same questions. (Supplementary Figure S5C,D). ABCD in Supplementary Figure S5 are shown as numerical values in Table 3. There was no significant difference in satisfaction with the interview by the doctors and nurses between the group using ARC sheets and the group not using ARC sheets. In other words, the mean values for the groups that used and did not use the ARC sheet were 3.16 and 3.48, respectively (*p* = 0.25), The results show that the group that received the pre-interview using the ARC sheet was slightly more satisfied with conducting the interview than the group that did not receive it (Figure 2A). Regarding the system for receiving pre-examination interviews, the average value for effective use of waiting time was low at 2.931; however, the average value for ease of pre-examination interviews was high at 4.217. The lower the CTCAE grade of each adverse event, the higher the satisfaction with the pre-examination interview. The adverse events with a high CTCAE grade and low satisfaction with pre-examination interviews were fatigue, peripheral neuropathy, and skin disorders (Figure 2B).

**TABLE 3:** Satisfaction of patients who attempted to report their adverse event to health care providers with the accuracy of the communication (*N* = 160).

Segments (average)	Items	Average of numerical values
Ease of communication (3.903)	A sense of security	3.87
	Ease of communicating the content of the consultation	4.031
	Degree of understanding	3.809
The quality of interviews and collaboration between healthcare professionals (3.845)	Accuracy of grasping changes in physical condition on the day	4.012
	Accuracy of grasping changes in physical condition until the day before	3.938
	Good information sharing between medical professionals	3.756
	Accuracy of grasping symptoms	3.772
	Satisfaction with the length of time supported	3.745
The nurse’s reaction and response during the interview (3.364)	Understanding potential symptoms	3.497
	Proposal of coping methods for adverse events	3.556
	Anxiety about not having a pre-examination interview	3.21
	Arrangement of symptoms to be communicated to doctors	3.611
The pre-examination interview system (3.552)	Effective use of waiting time	2.931
	Feel free to have a pre-examination interview	4.217
	Privacy considerations	3.509
The entire segment		3.714

### Differences in Adverse Event Characteristics Between ARC Sheet Immune Checkpoint Inhibitors and Chemotherapy/Molecular Targeted Therapy Combinations

**FIGURE 3:**
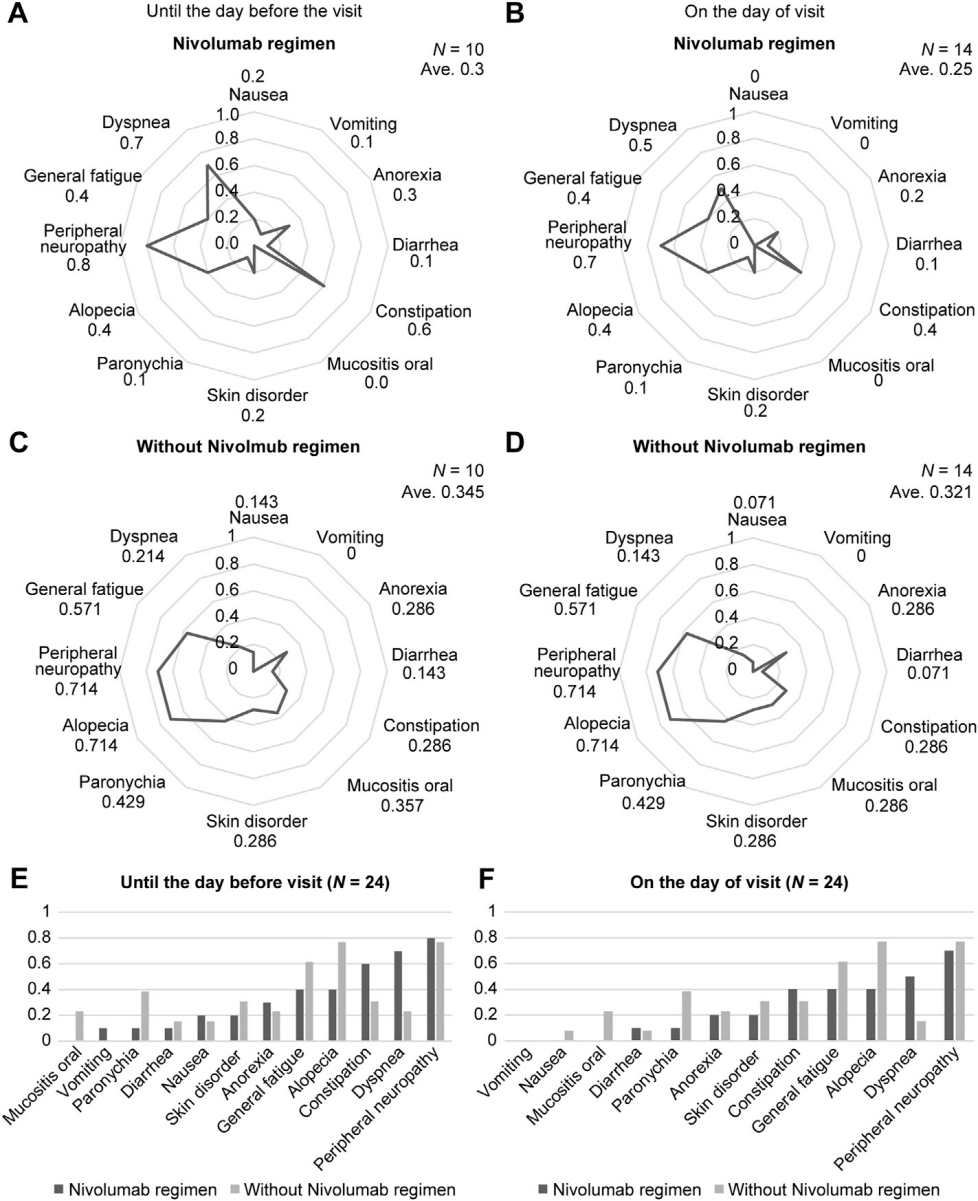
The results for the ARC sheet use group are shown. Assessment and risk control (ARC) sheets reflect differences in adverse events according to drugs used. **(A)**: Mean grade of CTCAE for each adverse event in the nivolumab use group up to the treatment date. Up to treatment day, patients in the nivolumab group had lower Common Terminology Criteria for Adverse Events (CTCAE) grades of oral mucositis, diarrhea, skin disorders, paronychia, hair loss, and general malaise than did those in the non-use group. **(B)**: Mean grade of CTCAE for each adverse event in the group using nivolumab on the day of treatment. On treatment day, patients in the nivolumab group had lower CTCAE grades of nausea, loss of appetite, oral mucositis, skin disorders, paronychia, hair loss, peripheral neuropathy, and general malaise than did those in the non-use group. **(C)**: Mean grade of CTCAE for each adverse event in the nivolumab-free group up to the treatment date. Up to treatment day, patients in the nivolumab-free group had a CTCAE grade mean for each adverse event compared to patients in the nivolumab-use group. **(D)**: Mean grade of CTCAE grade for each adverse event in the nivolumab-free group on treatment day. At the treatment date, patients in the nivolumab-free group had a higher mean CTCAE grade for each adverse event. **(E)**: Comparison of the mean CTCAE grade for each adverse event between the nivolumab-using and non-nivolumab-using groups up to the date of treatment. The severity of mouth ulcers and paronychia up to treatment day is significantly different between patients who received nivolumab and those who did not. **(F)**: Comparison of the mean grade of CTCAEs for each adverse event between the nivolumab-using and non-nivolumab-using groups on the day of treatment. The severity of mouth ulcers and paronychia on treatment day is significantly different between patients who received nivolumab and those who did not.

We next examined the usefulness of combining ARC sheets with immune checkpoint inhibitors and chemotherapeutic agents/molecular target drugs—an approach used increasingly in recent years—using esophageal cancer as an example. Nivolumab accounted for nearly half of the drugs used in patients with esophageal cancer, followed by paclitaxel, fluorouracil, and cisplatin (Supplementary Figure S7). Immune checkpoint inhibitors and chemotherapies/molecularly targeted therapies differ in adverse event characteristics; therefore, separate analyses were performed for patients who received nivolumab and those who did not. The use of nivolumab was associated with a lower mean CTCAE grading of 0.3 and 0.25 when compared to the day of treatment and between the last treatment and the current treatment, respectively. When the mean CTCAE grades for each symptom were compared between the nivolumab-free and nivolumab-use groups, there was no significant difference in any of the symptoms (Figures 3A–D). Until the day before the treatment, patients receiving nivolumab had lower CTCAE grades of oral mucositis, diarrhea, skin disorders, paronychia, hair loss, and general fatigue than those who did not receive nivolumab (Figure 3A,C). On the day of treatment, patients receiving nivolumab had lower CTCAE grades of nausea, loss of appetite, aphthous ulcer, skin disorders, paronychia, hair loss, peripheral neuropathy, and general fatigue than those who did not receive nivolumab (Figure 3B,D). The mean CTCAE grades of oral mucositis (*p* = 0.027 vs 0.02) and paronychia (*p* = 0.033 vs 0.033) up to and on the day of treatment were significantly different between patients who received nivolumab and those who did not receive nivolumab (Figure 3E,F; Supplementary Tables S4, S5).

## Discussion

ARC sheets were helpful to understand the adverse events of aphthous ulcers and dysgeusia and increase nurses’ sense of security. We found that the lower the mean CTCAE grade of each adverse event, the higher nurses’ satisfaction with the pre-examination interview. When analyzed by the difference in the drugs used for patients with esophageal cancer, the nivolumab group had lesser severity of aphthous ulcers and paronychia than did the chemotherapy group, and a significant difference was observed.

Aphthous ulcers manifest as erythema, swelling, and ulceration, doubling the likelihood of discontinuation of cancer drug therapy (Chaveli-López, 2014; Elad et al., 2020). Dysgeusia is observed in 46–77% of patients on cancer medication and can reduce dietary satisfaction and inadequate nutritional intake (Bernhardson et al., 2008; Zabernigg et al., 2010; Amézaga et al., 2018). Moreover, it is a symptom easily overlooked by patients or medical staff (Zabernigg et al., 2010). It was revealed that using the ARC sheet is beneficial in understanding the symptoms of aphthous ulcers and dysgeusia. Oral mucositis and dysgeusia are interrelated. Appropriate evaluation, prevention, and early detection, and symptom relief of these adverse events are thought to lead to maintenance of quality of life (QOL) and continuation of cancer drug therapy.

Obtaining information to be placed on patients’ medical records is considered a time-consuming task. Nevertheless, according to our study’s results, the symptoms of CTCAE grades 0–1 could be fully determined using the ARC sheet. Therefore, it is suggested that an ARC sheet, in which observation items are defined in advance, can comprehensively evaluate adverse events and facilitate and improve the efficiency of information sharing. However, the problem with the ARC sheet is that it does not have a formatted way to describe when the adverse event occurred or how the grade of the adverse event changed from the time of the previous chemotherapy to the day of the event. This is because there was no significant difference between the average grade evaluation of adverse events from the time of the previous chemotherapy to the previous treatment day and the average grade evaluation of adverse events on the treatment day. Therefore, details of the grade evaluation for adverse events from the time of the previous chemotherapy to the day of the treatment should be added to the ARC sheet’s observation items, together with the time of occurrence.

Pre-examination interview by nurses familiar with our anticancer drug therapy showed a high level of comfort with the nurse’s interview, although there was no significant difference between patients with and without the use of ARC sheets. Patients were generally satisfied that it was easier to report adverse events during the pre-test interview conducted by the nurse. It is noteworthy that the ARC sheet utilization group was pleased with the results regarding the nurses’ sense of security. However, there was no significant difference between the ARC sheet utilization group and the non-utilization group in terms of overall satisfaction. The rate of agreement between the two parties in the assessment of adverse events was lower between patients and physicians than between physicians and nurses or between patients and nurses. Agreement in symptom assessment was also highest between nurses and 11 reported that the PRO-CTCAE can be used to improve quality of life (Cirillo et al., 2009).

However, in this study, the obtained results for QOL after 1 month were better when pre-examination interviews, including CTCAE evaluation by specialized nurses, were added than when PRO-CTCAE was used alone (Basch et al., 2017; Baratelli et al., 2019). In general, Japanese patients face the dilemma of being unable to convey what they want to convey within a limited consultation time because they are reluctant to be examined by physicians. Consequently, physicians may think that a patient is “symptom-free” because they do not report any symptoms during the examination (Okamoto, 2007). Therefore, giving patients the opportunity to convey adverse events through pre-examination interviews with nurses has the advantage of allowing objective evaluation of adverse events in patients too anxious or tense to disclose them to physicians. In addition, interviewing the patient before the consultation helps to organize the information needed during the consultation, to properly understand the patient’s pain, and to allow the patient to properly communicate with the doctor regarding what they want to communicate.

In the present study, there was a low degree of patient satisfaction with the questionnaire survey in terms of smooth communication between patient and physician regarding adverse events with a high average CTCAE grade, such as fatigue, peripheral neuropathy, and skin disorders. Previous studies have shown that physicians underestimate fatigue, pain, constipation (Basch et al., 2006; Basch et al., 2009; Laugsand et al., 2010), and anxiety, indicating a greater tendency to overlook patients’ adverse events resulting from cancer medication (Basch, 2010). Moreover, our results showed that when the fatigue level was high, patients had low satisfaction with the transmission of information concerning adverse events. For patients, gastrointestinal symptoms such as nausea are easily reported in association with adverse events of cancer drug therapy. Therefore, physicians easily identify them as adverse events of cancer drug therapy. Peripheral neuropathy and malaise are difficult to judge visually and are easily underestimated. Therefore, the ARC sheet may be useful as a comprehensive sheet that includes an assessment of potential adverse events. However, it was found that the level of satisfaction with the questionnaire using the ARC sheet for these symptoms was low. We believe this is not because of dissatisfaction with the ARC sheets themselves, but because there is no quick-acting, evidence-based treatment for the peripheral neuropathy and fatigue discussed here.

Until recently, the standard treatment for advanced esophageal cancer has mainly consisted of fluorouracil, platinum preparations, and taxane-based cancer drug therapy combined with radiation therapy (Zhao et al., 2019). Recently, immune checkpoint inhibitors have been developed as second-line treatments for advanced, recurrent esophageal cancer (Watanabe, 2018; Hirano and Kato, 2019; Zhao et al., 2019). Immune-related adverse events of immune checkpoint inhibitors can occur widely from early to late dosing and require careful monitoring and timely management (Kato et al., 2019). In our study, there was no difference in the graded mean of CTCAEs over time for adverse event symptoms on the ARC sheet, despite the fact that the drugs have very different adverse event characteristics: cell-killing anticancer drugs and immune checkpoint inhibitors. This may be due to the fact that no immune-related adverse events occurred within the time period studied in the group that used nivolumab, and it is possible that the investigators evaluated adverse events of the drug used as primary treatment or physical symptoms arising from esophageal cancer itself. However, the ARC sheet may only partially cover the symptoms of immune-related adverse events. Therefore, we thought it necessary to identify the symptoms of relatively frequent immune-related adverse events and add the symptoms of these frequent immune-related adverse events as an observation item to the ARC sheet. Regarding individual adverse events, the severity of aphthous ulcer and paronychia was lower in the nivolumab use group than in the non-use group, and the difference between the two groups was significant. An aphthous ulcer is an adverse event that occurs in 20–40% of patients receiving cell-killing cancer drug therapy (Lalla et al., 2014). Therefore, the appearance of aphthous ulcers due to cell-killing cancer drug therapy used in the first-line treatment leads to further acceleration of the original weight loss and deterioration of nutritional status (Anandavadivelan and Lagergren, 2016). Therefore, using the ARC sheet to detect oral mucositis at an early stage and to initiate early treatment will improve patients’ nutritional status. Paronychia is presumed to have emerged as an adverse event in taxane-based cancer drug therapy, which is a third-line treatment.

Pre-visit interview by nurses using ARC sheets can be a tool to provide more comfort to patients who struggle to communicate complex adverse events to doctors in a straightforward and accurate manner. However, it was reported that there is more discrepancy in the evaluation of subjective adverse events than objective adverse events between medical professionals and patients (Basch et al., 2009). In order to reduce the disadvantages of the ARC sheet, we have made two efforts in the nurses’ pre-visit interview. The first is to avoid bias in the nurse’s subjective evaluation by using the diary of adverse events written by the patient each time. The second is for the physician to discuss with the nurse in charge of the interview the adverse events that have a high CTCAE grade, and to focus on the patient’s symptoms. We thought that these efforts will help ensure the quality of medical care and the safe and effective implementation of cancer drug therapy through the use of ARC sheets by nurses in the pre-visit interview.

### Strength and Limitation

The main strength of this study is that we were able to ascertain the usefulness of using the face-to-face ARC sheet to understand adverse events according to the checklist and share details immediately with the medical team. Conversely, the study design did not enable comparison of whether patient satisfaction was higher with or without the ARC sheet being used.

## Conclusion

In conclusion, the ARC sheet is a comprehensive tool used to evaluate potential adverse events and understand the adverse events of aphthous ulcers and dysgeusia. Moreover, patients reported a high sense of security in the pre-examination interviews by nurses using the ARC sheet. However, the ARC sheet had the following main limitation: the method of describing the time of occurrence of adverse events and the transition of grade from the time of the previous chemotherapy to the day of chemotherapy was not formatted. In the future, the need to add the details of the grade evaluation of adverse events up to the day of treatment to the observation items on the ARC sheet along with the time of occurrence will remain an issue.

## Data Availability

The original contributions presented in the study are included in the article/Supplementary Material, further inquiries can be directed to the corresponding author.
