# Supplementary figures and images for: Verification of the Usefulness of an Assessment and Risk Control Sheet that Promotes Management of Cancer Drug Therapy
Source: Front Pharmacol. 2022 Feb 9;13:744916. doi: 10.3389/fphar.2022.744916 (PMC8864067; doi:10.3389/fphar.2022.744916)

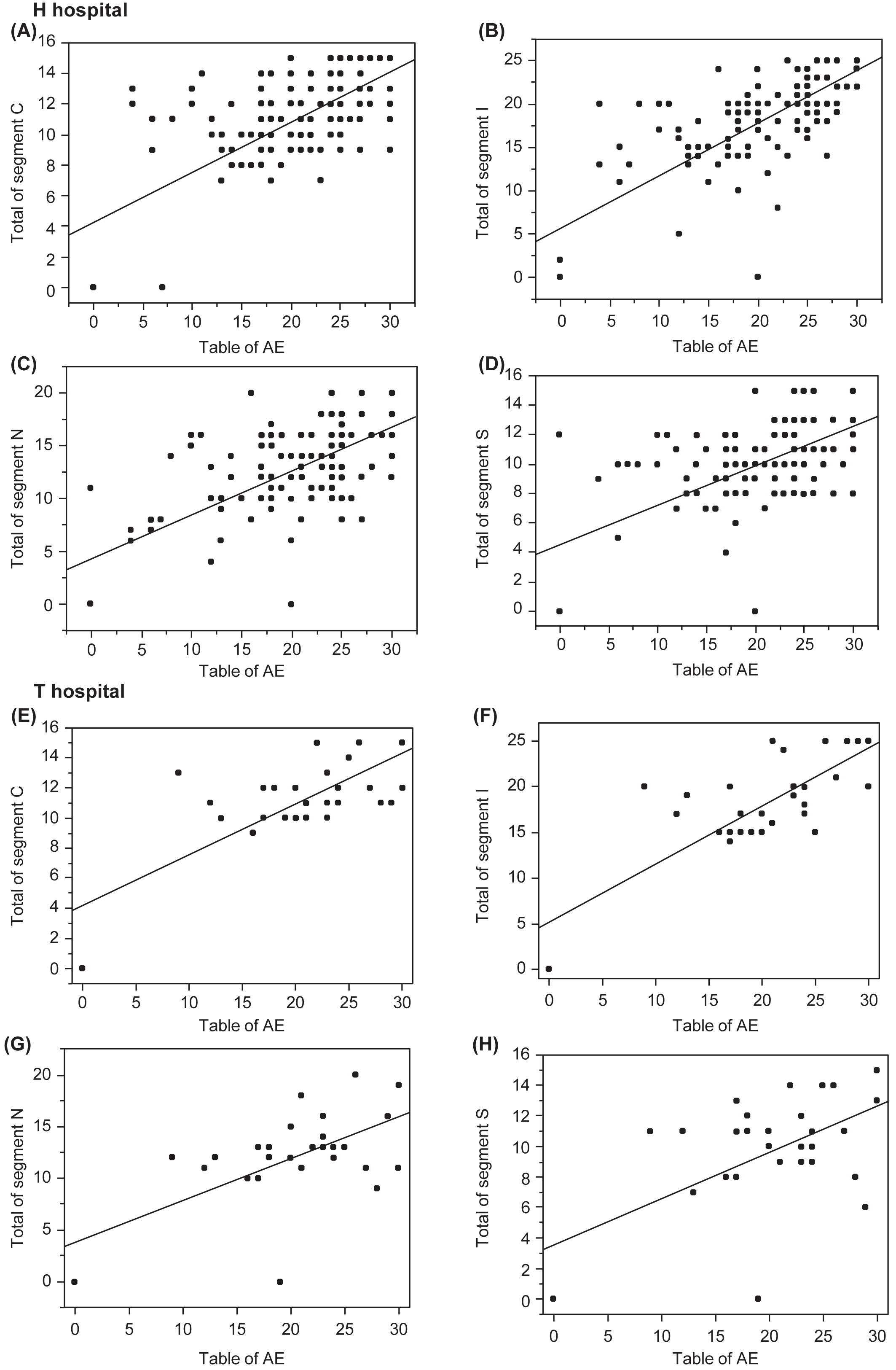

Supplement: Supplementary file 1 [file Image6.TIF]

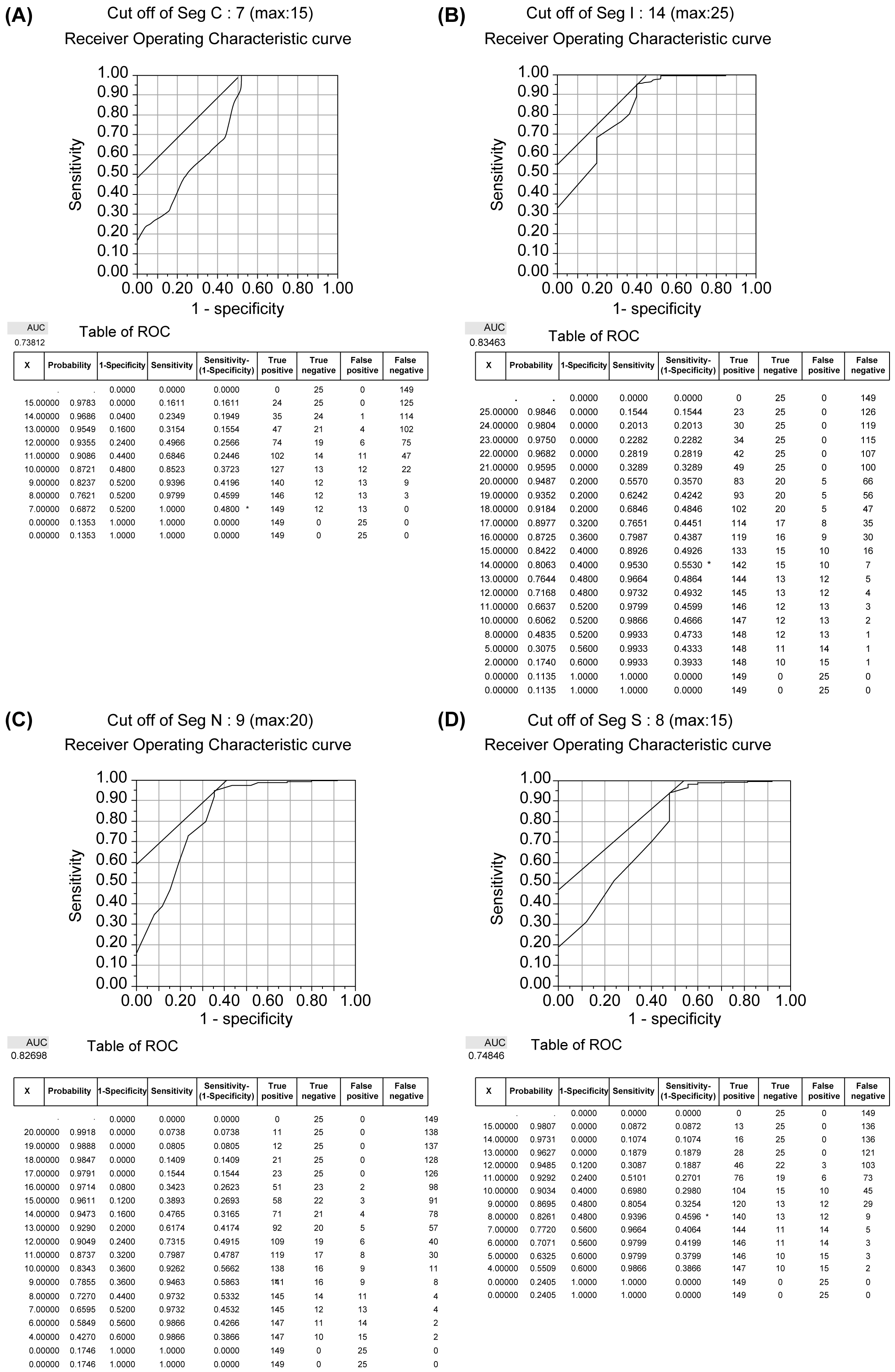

Supplement: Supplementary file 2 [file Image3.TIF]

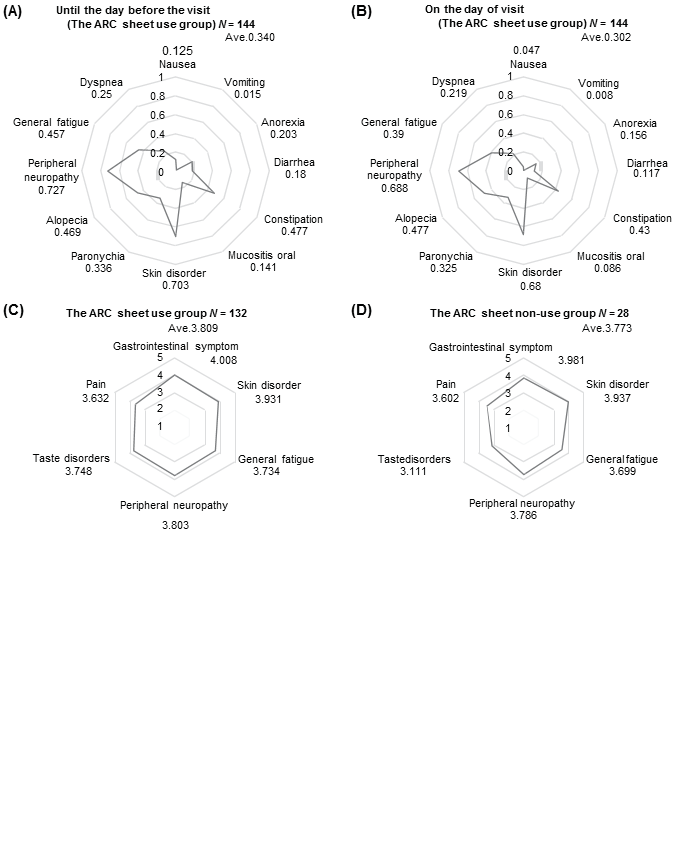

Supplement: Supplementary file 3 [file Image4.TIF]

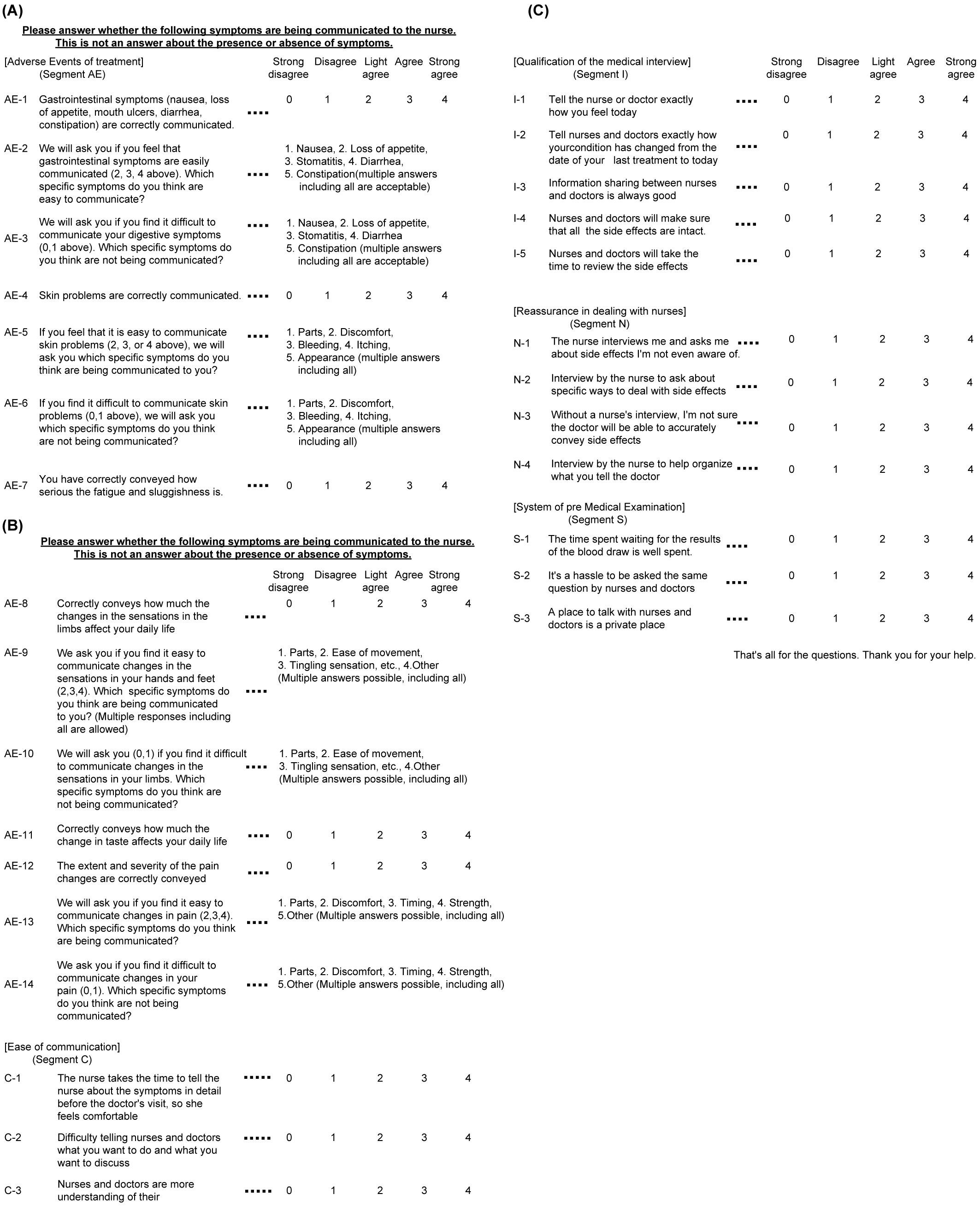

Supplement: Supplementary file 4 [file Image2.TIF]

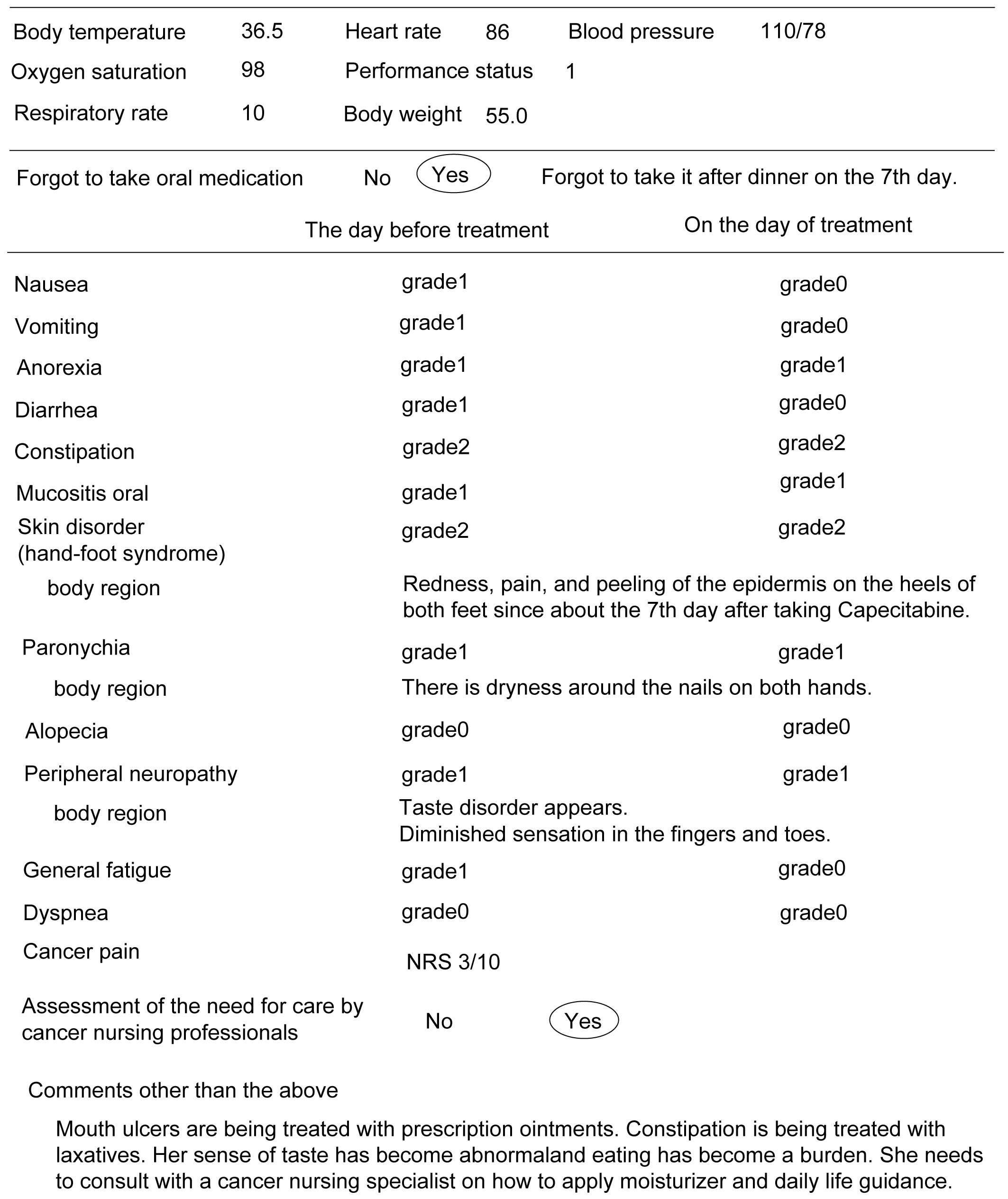

Supplement: Supplementary file 5 [file Image1.TIF]

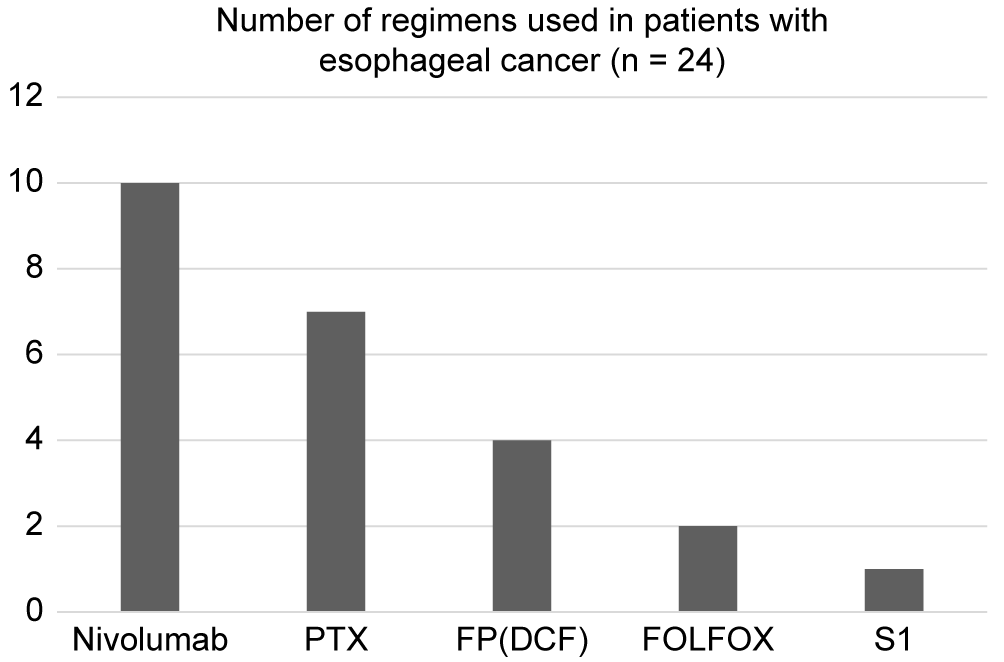

Supplement: Supplementary file 6 [file Image7.TIF]

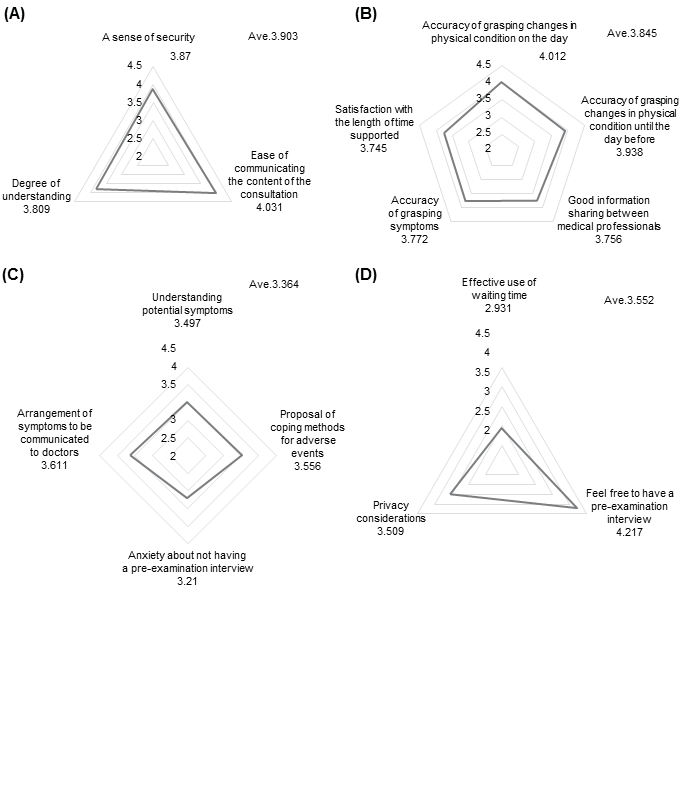

Supplement: Supplementary file 7 [file Image5.TIF]
