# Supplementary material for: Verification of the Usefulness of an Assessment and Risk Control Sheet that Promotes Management of Cancer Drug Therapy
Source: Front Pharmacol. 2022 Feb 9;13:744916. doi: 10.3389/fphar.2022.744916 (PMC8864067; doi:10.3389/fphar.2022.744916)
Supplement: Supplementary file 8 [file DataSheet1.docx]

| **Tumor (Bold) /**Regimens | Number | **Tumor (Bold) /**Regimens | Number | | **Tumor (Bold) /**Regimens | Number |
| --- | --- | --- | --- | --- | --- | --- |
| **GIST** | **1** | **Esophageal cancer** | **24** | | **Breast cancer** | **12** |
| Sunitinib | 1 | FOLFOX | 2 | | BV+PTX | 1 |
| **Ewing's sarcoma** | **1** | 5-FU+CDDP(DCF) | 4 | | DTX | 1 |
| VDC-IE | 1 | Nivolumab | 10 | | PER+HER+DTX | 1 |
| **Malignant Melanoma** | **1** | PTX | 7 | | PER+HER+Nab-PTX | 1 |
| Nivolumab | 1 | S-1 | 1 | | PER+HER+PTX | 1 |
| **Gastoric cancer** | **23** | **Neuroendoctine Tumors** | **1** | | S-1 | 1 |
| Capecitabine+CDDP+Tmab | 1 | Everolimus | 1 | | VNR+HER | 1 |
| Capecitabine | 1 | **Colorectal cancer** | **1** | | Abemaciclib+Leuprorelin | 1 |
| CPT-11 | 2 | FOLFOX+Panitumumab | 1 | | Palbociclib＋Fulvestrant | 1 |
| FOLFOX | 3 | **Gallbladder cancer** | **1** | | Trastuzumab Emtansine | 2 |
| Nab-PTX | 3 | GEM+CDDP | 1 | | Abemaciclib+Fulvestrant | 1 |
| Nivolumab | 3 | **Neuroendoctine Tumors of rectum** | **1** | | **Lung cancer** | **30** |
| PTX | 1 | Capecitabine+L-OHP | 1 | | CBDCA+ETP+Atezolizumab | 1 |
| Ramucirumab+PTX | 1 | **Rectum cancer** | **19** | | CBDCA+Pemetrexed | 1 |
| S-1 | 5 | Capecitabine+BV | 1 | | CDDP+Pemetrexed+Pmab | 1 |
| S-1+L-OHP | 1 | Capecitabine | 1 | | DTX | 1 |
| TAS-102 | 2 | Capecitabine+L-OHP+Bev | 1 | | Nivolimab | 5 |
| **Colon cancer** | **16** | FOLFIRI+BV | 3 | | Pemetrexed+BV | 1 |
| Capecitabine+BV | 2 | FOLFOX+BV | 1 | | S-1 | 2 |
| CPT-11 | 1 | FOLFOX+Panitumumab | 4 | | Atezolozumab | 2 |
| FOLFIRI | 1 | S-1 | 1 | | Afatinib | 4 |
| FOLFIRI+Cetuximab | 1 | S-1+BV | 1 | | Osimertinib | 3 |
| FOLFOX+Panitumumab | 3 | TAS-102+BV | 1 | | Crizotinib | 1 |
| FOLFOXIRI | 1 | XELOX | 3 | | Gefitinib | 1 |
| Panitumumab | 1 | CPT-11＋Panitumumab | 1 | | Durvalimab | 2 |
| TAS-102 | 2 | Regorafenib | 1 | | Pembrolizumab | 4 |
| TAS-102+BV | 1 | **Smooth muscle sarcoma** | **4** | | Lorlatinib | 1 |
| UFT/UZEL | 1 | Eribulin | 4 | | **Pancreatic cancer** | **1** |
| CPT-11＋Panitumumab | 1 | **Appendiceal cancer** | **1** | | PTX+GEM | 1 |
| Regorafenib | 1 | BV+FOLFIRI | 1 | |  |  |
| **Carcinoma of unknown primary** | **2** | **Ovarian cancer** | **3** | |  |  |
| GEM | 1 | Maintenance for BV | 1 | |  |  |
| DXR | 1 | CDDP+DTX | 1 | |  |  |
| **Small intestine GIST** | **1** | PTX+BV | 1 | |  |  |
| Sunitinib | 1 | **Bladder cancer** | **1** | |  |  |
| CBDCA+GEM | 1 |  |  |  |  |  |

(*N*=144)

Supplementary Table 1

Supplementary Table 2 (*N*=30)

| **Tumor（Bold）/**Regimens | Number |
| --- | --- |
| **Gastric cancer** | **5** |
| Ramucirumab+nab-PTX | 1 |
| S-1 | 2 |
| S1+DTX | 1 |
| S-1+L-OHP+Ramucirumab | 1 |
| **Colon cancer** | **13** |
| CPT-11+Panitumumab | 1 |
| FOLFOX6 | 1 |
| FOLFOXIRI | 1 |
| FOLFOXIRI+BV | 1 |
| S1+CPT-11+BV | 1 |
| Capecitabine+CPT-11+BV | 3 |
| Capecitabine+BV | 1 |
| S-1+BV | 1 |
| Capecitabine | 1 |
| Regorafenib | 1 |
| Regorafenib＋BV | 1 |
| **Duodenal cancer** | **1** |
| S-1 | 1 |
| **Gallbladder cancer** | **1** |
| GEM+CDDP | 1 |
| **Rectal cancer** | **10** |
| S-1 | 1 |
| S-1+CPT-11+BV | 2 |
| Capecitabine+L-OHP | 2 |
| Capecitabine＋BV | 1 |
| Cetuximab | 1 |
| Regorafenib＋BV | 3 |

Supplementary Table 3 (*N*=144)

| Type of toxicity | The day before the treatment | | | | | On the day of treatment | | | |
| --- | --- | --- | --- | --- | --- | --- | --- | --- | --- |
|  | Grade0 | Grade1 | Grade2 | Grade3 |  | Grade0 | Grade1 | Grade2 | Grade3 |
| Nausea | 125 (87) | 19 (13) | 0 | 0 |  | 137 (95) | 7 (5) | 0 | 0 |
| Vomiting | 141 (98) | 3 (2) | 0 | 0 |  | 143 (99) | 1 (1) | 0 | 0 |
| Anorexia | 113 (78) | 29 (20) | 2 (1) | 0 |  | 120 (83) | 22 (15) | 2 (1) | 0 |
| Diarrhea | 119 (83) | 24 (17) | 1 (1) | 0 |  | 127 (88) | 17 (12) | 0 | 0 |
| Constipation | 96 (67) | 28 (19) | 20 (14) | 0 |  | 101 (70) | 26 (18) | 17 (12) | 0 |
| Mucositis oral | 124 (86) | 18 (13) | 2 (1) | 0 |  | 131 (91) | 12 (8) | 1 (1) | 0 |
| Skin disorder | 61 (42) | 74 (51) | 8 (6) | 1(1) |  | 63 (44) | 73 (51) | 7 (5) | 1 (1) |
| (hand-foot syndrome) |  |  |  |  |  |  |  |  |  |
| Paronychia | 98 (68) | 43 (30) | 3 (2) | 0 |  | 100 (69) | 40 (28) | 3 (2) | 0 |
| Alopecia | 97 (67) | 22 (15) | 25 (17) | 0 |  | 96 (67) | 23 (16) | 25 (17) | 0 |
| Peripheral neuropathy | 56 (39) | 71 (49) | 17 (12) | 0 |  | 62 (43) | 65 (45) | 17 (12) | 0 |
| General fatigue | 79 (55) | 61 (42) | 3 (2) | 0 |  | 88 (61) | 53 (37) | 3 (2) | 0 |
| Dyspnea | 106 (74) | 36 (25) | 2 (1) | 0 |  | 112 (78) | 30 (21) | 2 (1) | 0 |

Data are number of patients (%)

Supplementary Table 4 (*N*=24)

| Type of toxicity | Regimen | Mean (SD) | *t* | *P* |
| --- | --- | --- | --- | --- |
| Nausea | Nivolumab | 0.2(0.422) | -0.3465 | 0.633 |
|  | Without Nivolumab | 0.143 (0.363) |  |  |
| Vomiting | Nivolumab | 0.1 (0.316) | -1 | 0.828 |
|  | Without Nivolumab | 0 |  |  |
| Anorexia | Nivolumab | 0.3 (0.483) | -0.06387 | 0.525 |
|  | Without Nivolumab | 0.286 (0.611) |  |  |
| Diarrhea | Nivolumab | 0.1 (0.316) | 0.24577 | 0.404 |
|  | Without Nivolumab | 0.143 (0.535) |  |  |
| Constipation | Nivolumab | 0.6 (0.843) | -1.00498 | 0.834 |
|  | Without Nivolumab | 0.286 (0.611) |  |  |
| Mucositis oral | Nivolumab | 0 | 2.109989 | 0.027* |
|  | Without Nivolumab | 0.357 (0.633) |  |  |
| Skin disorder | Nivolumab | 0.2 (0.422) | 0.468 | 0.322 |
|  | Without Nivolumab | 0.286 (0.469) |  |  |
| Paronychia | Nivolumab | 0.1 (0.326) | 1.934839 | 0.033* |
|  | Without Nivolumab | 0.429 (0.514) |  |  |
| Alopecia | Nivolumab | 0.4 (0.843) | 0.835 | 0.206 |
|  | Without Nivolumab | 0.714 (0.994) |  |  |
| Peripheral neuropathy | Nivolumab | 0.8 (0.919) | -0.25712 | 0.599 |
|  | Without Nivolumab | 0.714 (0.611) |  |  |
| General fatigue | Nivolumab | 0.4 (0.516) | 0.803626 | 0.215 |
|  | Without Nivolumab | 0.571 (0.514) |  |  |
| Dyspnea | Nivolumab | 0.7 (0.675) | -2.00806 | 0.967 |
|  | Without Nivolumab | 0.214 (0.426) |  |  |

Supplementary Table 5(*N*=24)

| Type of toxicity | Regimen | Mean (SD) | *t* | *P* |
| --- | --- | --- | --- | --- |
| Nausea | Nivolumab | 0 (0) | 1 | 0.633 |
|  | Without Nivolumab | 0.071 (0.267) |  |  |
| Vomiting | Nivolumab | 0 | ― | ー |
|  | Without Nivolumab | 0 |  |  |
| Anorexia | Nivolumab | 0.2 (0.422) | 0.406 | 0.344 |
|  | Without Nivolumab | 0.286 (0.611) |  |  |
| Diarrhea | Nivolumab | 0.1 (0.316) | -0.2325 | 0.590 |
|  | Without Nivolumab | 0.071 (0.535) |  |  |
| Constipation | Nivolumab | 0.4 (0.843) | -0.41572 | 0.658 |
|  | Without Nivolumab | 0.286 (0.611) |  |  |
| Mucositis oral | Nivolumab | 0 | 2.280351 | 0.02* |
|  | Without Nivolumab | 0.286 (0.469) |  |  |
| Skin disorder | Nivolumab | 0.2 (0.422) | 0.468472 | 0.322 |
|  | Without Nivolumab | 0.286 (0.469) |  |  |
| Paronychia | Nivolumab | 0.1 (0.326) | 1.934839 | 0.033* |
|  | Without Nivolumab | 0.429 (0.514) |  |  |
| Alopecia | Nivolumab | 0.4 (0.843) | 0.834749 | 0.206 |
|  | Without Nivolimab | 0.714 (0.994) |  |  |
| Peripheral neuropathy | Nivolumab | 0.7 (0.823) | 0.04648 | 0.481 |
|  | Without Nivolimab | 0.714 (0.611) |  |  |
| General fatigue | Nivolumab | 0.4 (0.516) | 0.803626 | 0.215 |
|  | Without Nivolumab | 0.571 (0.514) |  |  |
| Dyspnea | Nivolumab | 0.5 (0.707) | -1.46514 | 0.916 |
|  | Without Nivolumab | 0.143 (0.363) |  |  |
